# Chronic wasting disease: a cervid prion infection looming to spillover

**DOI:** 10.1186/s13567-021-00986-y

**Published:** 2021-09-06

**Authors:** Alicia Otero, Camilo Duque Velásquez, Judd Aiken, Debbie McKenzie

**Affiliations:** 1 Department of Biological Sciences, University of Alberta, Edmonton, AB Canada; 2 Centre for Prions and Protein Folding Diseases, University of Alberta, Edmonton, AB Canada; 3 Department of Agricultural, Food and Nutritional Sciences, University of Alberta, Edmonton, AB Canada; 4 Present Address: Centro de Encefalopatías y Enfermedades Transmisibles Emergentes, Universidad de Zaragoza, Zaragoza, Spain

**Keywords:** Chronic wasting disease, prion, prion disease, prion pathogenesis, interspecies transmission

## Abstract

The spread of chronic wasting disease (CWD) during the last six decades has resulted in cervid populations of North America where CWD has become enzootic. This insidious disease has also been reported in wild and captive cervids from other continents, threatening ecosystems, livestock and public health. These CWD “hot zones” are particularly complex given the interplay between cervid *PRNP* genetics, the infection biology, the strain diversity of infectious prions and the long-term environmental persistence of infectivity, which hinder eradication efforts. Here, we review different aspects of CWD including transmission mechanisms, pathogenesis, epidemiology and assessment of interspecies infection. Further understanding of these aspects could help identify “control points” that could help reduce exposure for humans and livestock and decrease CWD spread between cervids.

## Introduction

Chronic wasting disease (CWD) is a highly prevalent prion disease affecting various species of the *Cervidae* family and has been described in North America, South Korea and Scandinavia [1, 2]. Prion diseases are fatal neurodegenerative disorders affecting numerous mammalian species. In addition to CWD, prion diseases include scrapie in sheep and goats, bovine spongiform encephalopathy (BSE), transmissible mink encephalopathy (TME), and Creutzfeldt-Jakob disease (CJD) in humans. CWD is the only prion disease affecting both wild and farmed animals and stands out for being highly contagious, widespread and persistent in the environment, which facilitates the transmission of the disease and hinders its control in deer populations [3–6].

Pathogenesis of CWD, as described for other prion diseases, occurs over extended asymptomatic periods and depends on the misfolding of the cellular prion protein (PrP^C^), encoded by the *PRNP* gene, into an infectious template-directing conformation (PrP^Sc^) [7]. Following exposure to prions, the host’s susceptibility to develop disease, the clinical presentation, and the neuropathology are regulated by the interaction between the host PrP^C^ primary structure and the invading prion agent or strain [8–10].

The neuropathology of affected cervids includes spongiform degeneration, neuronal loss, gliosis, and accumulation of PrP^CWD^ (cervid PrP^Sc^) in the form of aggregates [3, 4, 11]. Variation in the disease presentation between cervids, including survival period, distribution of brain lesions and PrP^CWD^ properties occur in concert with different prion strains [12–15]. Prion strains are reproducible biological information encoded in specific PrP^CWD^ conformers that are replicated by the templated-misfolding of the host PrP^C^ [16–18]. While the transmission of a prion strain between hosts sharing similar PrP^C^ is more efficient given the compatibility of selected strain-specific PrP^Sc^ conformers, transmission between hosts species expressing different PrP^C^ primary structures is relatively inefficient and can introduce permanent conformational modifications resulting in the emergence of strains with novel properties [14, 19–21]. Alternatively, a strain can be transmitted back and forth between two species expressing various PrP^C^ amino acid differences and remain unaltered informationally [22–24]. Strain selection from a host co-infected with multiple strains can also occur following transmission between species expressing different PrP^C^ molecules [12, 25]. In addition, some tissues may show differential susceptibility for some strains compared to the brain (e.g. spleen) [26].

The transmission cycle of CWD in wild and captive cervids also involves the propagation of prion strains within and between various host species expressing distinct PrP^C^ primary structures [12, 27, 28]. As CWD outbreaks become enzootic in cervid populations, circulating CWD strains must adapt to the shifting PrP^C^ landscape that each new host provides which might result in novel strain emergence [13, 14, 21, 29]. These shifts in the prion replication substrate (PrP^C^) also occur at the population level, with allele frequencies of protective PrP^C^ polymorphisms increasing in response to CWD in deer and elk [27, 30]. Here, we review the current understanding of the CWD transmission cycle, pathogenesis, infection biology and infer from numerous bioassay studies the potential for transmission to other species, including humans.

## Transmission of CWD

The progression of CWD is less understood in wild free-ranging cervids given its direct relationship with the infectious dose, the route of exposure, the prion strain and the host *PRNP* genotype. Under controlled conditions, the incubation period (i.e., time to onset of clinical signs) of experimentally infected, orally dosed white-tailed deer, mule deer and reindeer expressing different PrP^C^ ranged between 1.5 and 6 years post-exposure [13, 31–33]. Similarly in elk, differences in incubation period were observed to range between 1.8 to 5.2 years depending on the elk *PRNP* genotype [34–36]. During the asymptomatic period, both captive and wild infected cervids contribute to the spread of CWD as they accumulate considerable amounts of prion infectivity throughout the body, which is shed through secretions and excretions into the environment [6, 37, 38].

The age range documented for cervids infected with CWD in captivity is fairly similar to that described for free-ranging animals [39, 40]. However, depending on the historical CWD prevalence and given the social nature of cervids it is possible to find pre-clinical CWD positive fawns (< 1 year-old animals) and yearlings (1–2 year old) [41]. The youngest identified clinically-positive free-ranging deer and elk were 16 and 21 months of age, respectively [4, 42]. An age range of 2.5–7.5 years has been reported for free ranging clinical deer and 1.8 to 10.5 years for elk [4]. More recently, evaluation of the prevalence of CWD infection by age as determined by detection of abnormal prion protein in 28 954 deer collected over seven years of surveillance in Wisconsin and Illinois enzootic zones reached similar conclusions as in the previous studies; the risk of CWD infection increases with age in both male and female deer (prevalence in adults 1.93%, yearlings 0.89 and 0.45% in fawns) [43]. Previous analyses of prevalence in a sample of 4510 deer culled within Wisconsin eradication zones, where CWD incidence has historically been and remains the highest, confirmed CWD infection in 3 to 3.4% of yearling deer irrespective of sex [44]. These findings suggest the higher prevalence in younger animals (yearlings and fawns) could be related to the extent to which CWD has been historically prevalent within a particular cervid population. The detection of prions in fawns could as well represent mother to offspring transmission [45].

Differences in age at which clinical CWD is observed can vary between species and are likely to depend on the source or origin of the infection. The first cases of CWD Scandinavian cervids, were described in various 9–16 year old moose, a 16 year-old red deer and 3–4 year old reindeer [2, 46, 47].

The prevalence of CWD in wild cervids also varies by sex. In CWD enzootic areas the incidence of infection is much higher in males than females [44, 48, 49]. Considering that no differences in susceptibility have been detected between captive male and female deer [3, 39, 40, 50, 51], the higher prevalence in free ranging males may be attributable to differences in behavior, particularly during the breeding season when males roam widely and interact with other males more avidly, increase the risk of contact with contaminated environments and infected animals [52, 53].

The clinical progression and signs of CWD in both captive and experimentally infected cervids vary within and between species [3, 13, 31]. A brief summary is included in Table 1. Initial clinical features are often subtle and transitory [13]. The most prominent clinical features include behavioral alterations and progressive deterioration of body condition (i.e., weight loss) that worsen over the course of weeks to months [54]. Altered postures with lowered head and ears, arching of the back and ataxia can also be displayed [13, 31, 42]. Advanced clinical disease may involve odontoprisis, polydipsia, polyuria, difficulty swallowing, regurgitation of rumen contents and excessive salivation with drooling. Following recumbency, aspiration pneumonia, dehydration, or hypothermia during the winter season (in wild affected animals) are the most likely causes of death [42]. Compared to deer, CWD-affected elk can present with nervousness and hyperesthesia and are more likely to display motor disturbances but less likely to develop polydipsia [54].

**Table 1:** **Summary of CWD presentation in different cervid species.**

Cervid species	Average age range clinical disease	Clinical signs	PrP^CWD^ in the brain	PrP^CWD^ in lymphoid tissues	References
Mule deer White-tailed deer	2.5–7.5 years	Poor body condition, altered postures, ataxia, odontoprisis, polydipsia, polyuria, drooling	Yes	Yes	[42, 54]
Elk	1.8–10.5 years	Poor body condition, nervoussness, hyperesthesia, ataxia	Yes	Yes	[84]
				No	[83]
Reindeer	3–4 years	Poor body condition, recumbency, lethargy	Yes	Yes	[2]
Moose	9–16 years	Poor body condition, reduced fear of humans	Yes	No	[46]
Red deer (Minnesota)	22 months	Recumbency	Yes	Yes	[156]
Red deer (Norway)	16 years	No clinical signs observed	Yes	No	[47]

Various factors contribute to the efficiency of the CWD transmission cycle. The primary mode of transmission between cervids, early in an outbreak, is likely by direct animal to animal interactions following exposure to an infected animal or environment [53, 55]. Although experimental infection in pregnant muntjac deer (*Muntiacus reevesi*) and detection of PrP^CWD^ in tissues from wild pregnant elk dams provide evidence for in utero transmission [45, 56], other studies indicate it plays a minor role in epidemiology compared to deer-to-deer transmission [57]. Given the rapid pre-clinical accumulation of PrP^CWD^ aggregates in lymphoid tissues associated with the alimentary and the intestinal mucosa, the oral route of infection is most likely [31, 58, 59]. However, inhalation of CWD-fomites has also been proposed as a mechanism of exposure [60]. The presence of CWD infectivity in antler velvet [61] leads to the question of whether prions can persist in the calcified antler and favor male to male intradermal inoculation during the rut, or represent a risk factor in terms of oral CWD transmission, as antler gnawing is common among cervids. Antler gnawing has been suggested as a factor involved in the transmission of CWD in the reindeer population from Nordfjella, Norway [62].

The spread of CWD into “naïve” cervids also occurs through exposure to contaminated environments previously inhabited by infected animals [38, 57, 63]. This mode of transmission becomes more relevant as the prevalence of CWD in affected cervid populations increases and the disease becomes enzootic. Secretions (saliva) and excretions (urine and feces) of CWD-infected cervids contain considerable CWD infectivity. The minimum infectious dose in saliva required for a deer to become infected, assuming a single oral exposure to CWD prions, is equivalent to the infectivity contained in 100–300 ng of brain (approximate equivalent of 30 mL of CWD-positive saliva) [6]. Secretions and excretions from CWD positive animals and the decomposition of diseased carcasses contaminate the environment, in which prions can persist in a bioavailable state for years [38, 64–66]. The physical association of prions with certain soil microparticles enhances transmissibility [67, 68]. Environmental persistence of CWD infectivity depends on the composition of minerals and organic constituents of soil, which vary between geographical areas [69]. Minerals such as montmorillonite, can enhance the experimental transmission of prions by the oral route [67]. Deer consume a significant amount of soil, especially in adjacent areas to mineral licks, which have tested positive for infectivity in CWD endemic regions [70]. Soils, depending on their composition, represent an important reservoir of CWD infectivity in the environment. Although determination of the degree of contamination of a particular soil surface becomes more difficult with time, the soil-bound prion infectivity is not significantly altered [66].

Plants can also represent an important reservoir for CWD contamination and transmission. CWD contaminated pastures can remain infectious for at least 2 years after prion exposure [65]. Regarding the uptake of prions by plants, results are more controversial. One study, using protein misfolding cyclic amplification (PMCA), an ultrasensitive technique for the detection of prions, demonstrated that grass plants exposed to brain or excretions from CWD-infected cervids can uptake prions from the soil and transport them to the aerial parts of the plant [71]. Another study, however, showed that wheat plants do not transport CWD prions from the roots to the stems [72].

## Prion neuroinvasion and body distribution of infectivity

The pathological hallmarks of CWD in deer resemble those observed in sheep with scrapie and other prion diseases acquired by ingestion of contaminated material. In orally infected deer, PrP^CWD^ crosses the intestinal epithelial barrier and can be detected, within the first 30–42 days post-exposure, in lymphoid tissues associated with the alimentary tract such as the gut-associated lymphoid tissue (GALT), the tonsils and the retropharyngeal lymph nodes [31, 59, 73, 74]. Modified enterocytes, called M cells, participate in the uptake of the prion, incorporating it into the subepithelial lymphoid tissue. The pathological prion protein then accumulates and replicates in follicular dendritic cells and tingible body macrophages [58, 75, 76]. PrP^CWD^ cellular targeting during early pathogenesis suggests that prions are transported by dendritic cells and/or macrophages to Peyer’s Patches and regional mesenteric lymph nodes [58, 59].

Once infection has been established in the GALT, prion colonization of the nerve endings of the Enteric Nervous System (ENS) and leakage into the lymph and blood facilitates the spread to other organs [77, 78]. Prion infection of the ENS results in the spread of infectivity through sympathetic and parasympathetic nerves [75]. The initial site of PrP^CWD^ detection within the deer brain is the dorsal motor nucleus of the vagus nerve (DMNV), suggesting this nerve as the major route for PrP^CWD^ traffic from the alimentary tract to the brain [73].

CWD infectivity trafficked via the lymph and the blood can reach multiple organs, including the brain. Prion neuroinvasion by this route likely occurs via the circumventricular organs [79]. Consistent with this observation, considerable prion infectivity has been demonstrated in numerous blood cell types from CWD-infected deer, suggesting that the haematogenous dissemination of infection may be important during the pathogenesis of the disease [80].

Another major route of prion neuroinvasion involving the entry via ENS is by retrograde transport of prions through the splanchnic nerve circuitry. This is consistent with the presence of prion aggregates in the intermediolateral columns of the thoracic spinal cord during early stages of prion infection [81, 82]. This route is particularly important during neuroinvasion by BSE prions in cattle, however, analysis of CWD prion accumulation following oral infection did not detect prion deposits in the coeliac ganglion of deer. This suggests that the accumulation of PrP^CWD^ in the intermediolateral column of orally infected cervids results from the centrifugal dissemination of prions replicated within the central nervous system (CNS) [73].

The early lymphoid replication phase is particularly important for the neuroinvasion of CWD prions in deer [73, 74]. Interestingly, North American elk as well as Scandinavian moose and red deer accumulate PrP^CWD^ in the brainstem with little to no accumulation in lymphoid tissues [46, 47, 83, 84]. This could be explained by a predominantly neural route of neuroinvasion (as in BSE pathogenesis), sporadic misfolding of PrP^C^ or differences in the route of exposure. Differences in pathogenesis and neuropathology of sheep inoculated via different routes were not seen [85]. Similarly, no significant differences have been detected in the peripheral burden of CWD prions from deer infected through different routes [64]. Once neuroinvasion has occurred, PrP^CWD^ accumulates producing the characteristic lesions of prion diseases, including intraneuronal vacuolation, neuropil spongiosis, gliosis and formation of amyloid plaques [86].

In addition to lymphoid and brain tissues, CWD prions have been detected in nasal mucosa, salivary glands, urinary bladder, pancreas, kidney, intestine and reproductive tract of female and male deer [15, 31, 64, 73, 87, 88]. The accumulation of PrP^CWD^ in some of these tissues is tightly associated with shedding of infectivity through secretions and excretions [64]. Similar to scrapie in sheep [89], *PRNP* genotype can influence CWD pathogenesis in deer affecting PrP^CWD^ deposition in peripheral tissues [13, 15, 31, 74, 90].

## CWD in cervids

The origin of CWD remains unknown. In North America, epidemiological data suggests emergence occurred in Colorado and Wyoming [55], in the late 1960s, in captive mule deer (*Odocoileus hemionus*) and black-tailed deer (*Odocoileus hemionus columbianus*) at research facilities. These herds were captured cervids from different wild populations, including pregnant females that were released after parturition. Transfer of animals between facilities was a common practice [3]. CWD was subsequently detected in Rocky Mountain elk (*Cervus elaphus nelsoni*) at these facilities and, thereafter, in free-ranging populations of mule deer and elk in Wyoming and Colorado [3, 50, 91].

Cervid migration and commercial movement of preclinical animals contributed to the geographic expansion of CWD into free-ranging and captive populations of North America [42, 55]. To date, CWD occurs in at least 26 U.S. states and three Canadian provinces (Saskatchewan, Alberta and Québec). In Canada, CWD was first identified in farmed elk from Saskatchewan in 1996 [42]. In the following years, CWD was reported in farmed white-tailed deer in Alberta and in wild cervid populations from Saskatchewan and Alberta [92]. Epidemiological studies suggested that the infection was introduced into Saskatchewan farms via import of captive elk from a farm in South Dakota [42]. The origin of the CWD epidemic in wild cervids of Canada remains unknown. Transmission by contact exposure between wild deer and infected farmed elk is a possibility [92]. CWD was first detected in 2013 in wild moose from Alberta [93]. CWD has not, to date, been detected in the wild North American subspecies of caribou (*Rangifer tarandus spp*).

Outside of North America, CWD outbreaks in captive cervids in South Korean farms occurred following cohabitation with asymptomatic infected elk and deer imported between 1994 and 2003 from a farm in Saskatchewan (later determined to house CWD-infected animals [94]. A direct consequence was the transmission into South Korean captive red deer (*Cervus elaphus*), sika deer (*Cervus nippon*) and crosses of these two species [28]. Epidemiological studies of CWD in wild cervids from the Korean peninsula are lacking.

In 2016, CWD was identified in a free-ranging Norwegian reindeer (*Rangifer tarandus tarandus*), representing the first CWD case detected in Europe [2]. Since this event, thousands of cervids have been surveyed, leading to the detection of the CWD in 19 reindeer, 4 moose and one red deer in Norway, 3 moose in Sweden and one moose in Finland, suggesting that CWD has been quietly emerging in European cervids [46, 47, 95]. The origin of these cases is still unknown. Transmission of Norwegian CWD isolates into bank voles demonstrated the presence of strains different than those seen in North America, suggesting these epizootics are not epidemiologically linked [96]. In addition, importation of cervids to Norway is not allowed and, therefore, it is unlikely that these CWD infections emerged from imported positive animals as was the case for CWD in Korea [1].

The prevalence of CWD in North America has been increasing exponentially during the last 6 decades. In farmed herds the prevalence of CWD positive animals can be higher than 80% and higher than 45% in wild populations [41]. In areas where CWD has become enzootic, the CWD prevalence can be greater than 50% in adult males [97]. The latest Alberta CWD surveillance update (2019 fall hunting season) indicates that the prevalence of CWD continues to increase in all cervid species. Compared to the 2018 fall hunting season, the prevalence in the 2019 season saw an increase of 3.8% (from 7.4% in 2018 to 11.2% 2019 prevalence). Consistent with previous years, white-tailed and mule deer in Alberta and Saskatchewan show differences in prevalence between species and sexes. The prevalence rank among Alberta deer is mule deer males > mule deer females > white-tailed males > white-tailed females. The burden of CWD in Alberta wild elk has not been as extensive as in deer. In 2019, 1.3% of the tested elk resulted positive for CWD (0.8% in 2018). In addition, for the first time, CWD was detected in two hunter-harvested moose [98].

Population declines are observed in cervid herds with high CWD prevalence. CWD positive deer not only succumb to the disease but are also more prone to be killed by predators or hunters, and are more vulnerable to vehicle collisions [99]. Average declines in elk survival in Rocky Mountain National Park were attributed almost entirely to CWD [100] Mean annual survival rates of CWD-negative and CWD-positive deer were estimated as 76% and 32%, respectively, and CWD was considered a significant contributor to mule deer population decline [101]. Miller et al. also observed that the 2-year survival of infected and uninfected tagged wild mule deer was 47 and 82%, respectively [99].

## Experimental CWD in cervid species

No natural cases of CWD have been described in some species of cervids, although they have proven to be susceptible to CWD following experimental exposure. These include the Asian muntjac (*Muntiacus reevesi*) [45] and fallow deer (*Dama dama*) [102]. Muntjac deer were successfully infected through oral and subcutaneous routes with CWD from white-tailed deer. Interestingly, PrP^CWD^ was detected in fetuses from CWD-infected does, demonstrating vertical CWD transmission in this species [45]. Although fallow deer were suggested to show certain resistance to infection with CWD [103], Hamir et al. reported that this species is susceptible to the disease after intracerebral inoculation with elk and white-tailed deer prions [102]. The differences in these studies may have arisen because intracranial inoculation is more efficient to produce disease compared to environmental exposure, however, PrP^C^ sequence and prion strain compatibility could also explain these differences [102, 103]. No natural cases of CWD have been reported in North American caribou, although these species are susceptible to experimental infection with CWD from mule deer, white-tailed deer and elk. Naive caribou can acquire the disease after oral infection [33] and environmental exposure [63]. A summary of this transmission experiments and the ones described below is included in Table 2.

**Table 2:** **Experiment﻿al transmission of CWD to different animal species.**

Species	Route of CWD transmission^a^	References
**Cervids**		
Muntjac deer	IC, PO, SC	[45]
Fallow deer	IC	[102]
North American caribou	PO, environmental	[33, 63]
**Livestock**		
Cattle	IC	[105, 108]
Sheep	IC	[110, 157]
Pigs	IC, PO	[116]
**Wildlife**		
** Rodents**		
Present in CWD endemic areas: Meadow voles Red-backed voles White-footed mice Deer mice House mice	IC	[29, 118]
Not present in CWD areas: Syrian golden hamster European bank vole	IC	[119, 120]
** Carnivores**		
Ferrets	IC, PO, IP	[121–123]
Mink	IC	[124]
Cats	IC, PO	[125]
Raccoons	IC	[128]

^a^IC: intracerebral, PO: oral, SC: subcutaneous, IP: intraperitoneal.

## Evaluating the potential transmission of CWD to non-cervid species

Most of the transmission studies of CWD into various animal species have been conducted with North American CWD isolates and have revealed different transmission patterns. The host range of European CWD isolates is still to be determined [95].

### Livestock species

The interactions between different animal species in captivity is a known factor favoring the emergence of new pathogens with novel zoonotic properties, as has been recently proposed as the origin of BSE in cattle by contact with sheep infected with atypical/Nor98 scrapie [104]. The distribution of CWD in North America could favor the interspecies transmission of cervid prions into cattle (i.e., overlapping of these species is common in CWD enzootic areas of North America). The transmission of a prion disease to the cattle is cause for alarm due to the potential emergence of BSE-like zoonotic capacity. CWD from different species (white-tailed deer, mule deer and elk) has been successfully transmitted to domestic cattle after intracerebral challenge with different attack rates [105–108]. The neuropathological and biochemical characteristics of bovine CWD are, however, clearly distinct from BSE [105]. In addition, oral infections in cattle with mule deer prions have been unsuccessful, and no positive transmission has been detected in this species after 10 years of environmental exposure to mule deer and elk CWD [109]. This demonstrates that an important species barrier limits the oral transmission of CWD to cattle.

In 2006, Hamir et al. reported the transmission of mule deer CWD into sheep via the intracranial route [110]. Only 2 of 8 inoculated lambs developed lesions compatible with a prion disease, and they expressed different *PRNP* genotypes at codons 136, 154 and 171, which are known to determine sheep susceptibility to scrapie [111–113]. One animal expressed ARQ/ARQ (subclinical) and one ARQ/VRQ (clinical) sheep. ARQ/ARR sheep were completely resistant to CWD inoculation, suggesting that the transmission of CWD to small ruminants is strongly determined by the host genotype, as seen with scrapie [110]. Clinical disease was described, however, in ARQ/ARQ sheep inoculated with elk CWD prions, which suggests a different strain in the elk isolate. Transgenic mice overexpressing the ovine VRQ PrP allele (tg338 mice) do not accumulate prions in the brain after experimental infection with a number of different CWD isolates [26, 114, 115]. These tg mice, however, efficiently replicate CWD prions in the spleen, suggesting that the lymphoid tissue is more permissive than the brain for interspecies transmission [26].

The susceptibility of pigs to CWD has also been investigated. Moore et al. found that white-tailed deer CWD prions can be detected by real-time quaking-induced conversion (RT-QuIC) in some orally and intracranially inoculated pigs when euthanized at market weight (8 months age, 6 months after inoculation). One aged pig showed clinical signs and, in these aged animals, PrP^CWD^ was detectable by immunohistochemistry and Western blotting in 4/10 intracranially inoculated and in 1/10 orally inoculated pigs. Passages in transgenic mice expressing the porcine PrP showed reduced attack rates. Therefore, they concluded that pigs could support a low-level propagation of CWD prions, albeit with a high species barrier. These results are not necessarily encouraging since it is possible that feral pigs, whose ranges are shared with CWD affected cervids, could act as a reservoir of CWD [116].

### Other wildlife species

Comparison of the *PRNP* sequences of different species of ungulates that inhabit CWD endemic areas showed high sequence identity between bighorn sheep (*Ovis canadensis*), mountain goats (*Oreamnos americanus*) and domestic sheep suggesting that these species are potentially susceptible to CWD [117]. No experimental challenges of these wildlife species, sympatric to deer in CWD-endemic areas have been performed yet.

#### Rodents

Several different rodent species sympatric with deer in CWD endemic areas including meadow voles (*Microtus pennsylvanicus*), red-backed voles (*Myodes gapperi*), white-footed mice (*Peromyscus leucopus*) and deer mice (*P. maniculatus*) have proven to be susceptible to CWD after experimental inoculation [118]. Among these species, meadow voles showed to be the most susceptible, but incubation periods were shortened in all the rodent species upon second passage, indicating CWD adaptation to these hosts. House mice (*Mus musculus*) live in close proximity to humans, and their susceptibility to particular CWD strains has been demonstrated [29]. It is possible that wild rodents represent a reservoir for CWD in ecosystems considering that these animals are scavengers, and one of the main sources of food for predators. In addition, they can be accidentally consumed by deer or livestock since rodent carcasses contaminate pastures and forage [118].

CWD is also transmissible to other rodent species that do not cohabitate with deer in CWD-affected regions. These include Syrian golden hamsters (*Mesocricetus auratus*) [119] and European bank voles (*Myodes glareolus*) [120]. Curiously, the adaptation of CWD to the European bank vole resulted in the identification of a prion strain (CWD-vole strain) with the shortest incubation period observed to date [120].

#### Carnivores

Ferrets (*Mustela putorius*) are a valuable model for the study of prions, including CWD [121–123]. Mink (*Mustela vison*) can also be infected with CWD, but only by intracerebral inoculation. The disease characteristics differed from those of TME-affected mink, demonstrating different strains cause CWD and TME, and suggest that mink are unlikely involved in natural CWD transmission [124].

Oral and intracerebral inoculation of mule deer prions into domestic cats (*Felis catus*) resulted in no clinical disease or low attack rates, respectively, on first passage. A second passage of the prions from the intracerebrally inoculated cats resulted in 100% of the recipient cats presenting with clinical disease while the second oral passage resulted in a 50% attack rate demonstrating the adaptability of CWD prions to felines [125]. The PrP^C^ sequence similarity between cats and mountain lions (*Puma concolor*) suggests that these wild carnivores would be susceptible to CWD infection [126]. As mountain lions selectively prey CWD-infected cervids, it would be of interest to test dead animals for CWD to evaluate for prion spillover [127].

CWD prions from white-tailed deer and elk transmitted with low attack rates (25%) following intracerebral challenge in raccoons (*Procyon lotor*) [128]. Accumulation of protease resistant prions in the cerebrum and obex differed depending on the inoculum. Interestingly, prions from mule deer did not transmit to raccoons after 6 years following intracerebral challenge [129, 130]. This further suggests strain differences in CWD prions from the various cervid species affected.

Among all mammals, canids are probably the most resistant to prion diseases, with the amino acid residue 163 of canine PrP^C^ conferring protection [131–133]. Oral exposure of captive coyotes (*Canis latrans*) to elk prions demonstrated the presence of prions in the coyote fecal material during the first days after consumption [134]. However, even after a large volume of infectious brain homogenate was inoculated, only 50% of exposed coyotes had detectable infectivity in feces between 1- and 4-days post-exposure (dpe) as evaluated by bioassay in tg12 mice (expressing elk PrP^C^), while the other half lacked detectable prions or were only recovered in feces after 1 day. No evidence of CWD accumulation in the coyote lymph tissue was detected [134]. These results suggest that coyotes were capable of degrading the CWD infectivity. Consistent with this interpretation, the attack rates were incomplete in tg12 mice inoculated with feces collected at various times following exposure. When inoculated with brain homogenates from CWD-infected elk, this transgenic mouse line develops prion disease with full attack rates after incubation periods of < 150 days post-infection [135]. In addition, in the wild, coyotes will likely consume a smaller infective dose as CWD infectivity in muscle and fat is lower than in the brain [51, 136]. The role of canine predators in the control of CWD has been discussed previously, suggesting that the selective predation exerted by wolves (*Canis lupus*), which hunt weak and vulnerable cervids, could represent an important natural tool to limit CWD contamination of the environment [137]. The reintroduction and protection of wolves in CWD-affected areas, although controversial, could be very efficient for the natural control of the disease.

### Humans

To date, there is no clear evidence that CWD can cross the transmission barrier and infect humans, as other animal prions such as BSE [138]. Several epidemiological studies have been developed to assess whether, statistically, there are more cases of prion diseases in population groups living in endemic areas for CWD. These studies mainly consider people exposed to CWD-infected cervids, such as consumers of deer meat and hunters. None of these studies have found a clear correlation between CWD exposure and an increase in human prion disease frequency [90, 139–141]. Evaluating the risk of humans to CWD through this type of studies is difficult due to the variety of strains present in the environment, the transport of hunted animals between long distances and the long incubation period of prion diseases in humans (even decades).The identification of the zoonotic ability of an agent requires an abnormally high number of human cases within a particular geographical location or period of time, which necessitates a large number of human exposures to the disease. Prevalence of CWD in several areas has increased exponentially in the last decade, therefore, there may not historically have been a sufficient level of exposure to the disease to detect a zoonotic transmission of CWD.

There are tools, however, to evaluate the susceptibility of humans to CWD. These include bioassays in non-human primates and transgenic mice expressing human PrP^C^ and in vitro studies of the human transmission barrier to CWD.

Squirrel monkeys (*Saimiri sciureus*) are susceptible to CWD prions from mule deer, elk and white-tailed deer after oral and intracerebral challenge [142, 143]. Race et al. did not observe evidence for CWD transmission to macaques (*Macaca fascicularis*) at 13 years post-inoculation and using ultra-sensitive techniques for the detection of prions [144]. In an ongoing study, macaques were exposed to different sources of CWD through various routes. Analysis of the tissues identified PrP deposition in the dorsal horns of the spinal cord in a subset of the macaques [145]. Similar PrP immunopositive staining affecting the spinal cord was also reported by Race et al.; these deposits were found in both CWD-challenged and uninoculated, aged macaques, suggesting that this staining was likely due to cellular PrP [144]. Evaluating the zoonotic potential to humans through bioassays in non-human primates has, however, several drawbacks. The degree of sequence similarity between human PrP and the PrP from non-human primates varies between 92.2 and 99.7% [146]. Even species with high sequence homology, such as chimpanzees, express amino acid substitutions in key structural motifs of PrP that could alter the transmission barrier of prions [146, 147]. Although chimpanzees are more closely related to humans, the presence of 2 amino acid polymorphisms adjacent and within the α2-β2 loop, an important structural motif modulating interspecies transmission of some prion strains [148], undermines their utility in testing the species barrier. In particular, the residue E168 in humans (Q168 in chimpanzees) appears to be fundamental for human reduced susceptibility to CWD and other prion strains from ruminants [149].

Transgenic mice expressing different variants of human PrP^C^ (MM129, MV129 and VV129) at 1–16-fold the levels expressed in the human brain were challenged with US and Canadian CWD isolates in seven different studies. Elegantly reviewed by Waddell et al., none of these studies found evidence of transmission to any of the transgenic mice (reviewed by [141]). Studies in chimeric mice suggested that the α2–β2 loop of the prion protein is the key to the transmission barrier of humans to CWD [148]. However, a recent study found low levels of RT-QuIC seeding activity in four mice overexpressing human prion protein (MM129) inoculated with elk and white-tailed deer isolates (2 mice per inoculation group). These results need to be interpreted with caution, as these reactions were inconsistently positive perhaps representing poorly adapted CWD prions into human PrP or alternatively, persistence of the inoculum in the brain of these mice since human prions can physically persist even in knock-out mice for extended periods post-inoculation [149]. In addition, these RT-QuIC positive mice (tg66) express the highest levels of human PrP tested in CWD transmission studies (8–16 × compared to human brain), which could facilitate the replication of CWD in human PrP [150]. In contrast, the same CWD isolates when inoculated into the tgRM (2–4×) resulted in less clinical suspects and no positive detection by RT-QuIC [150].

Finally, the transmission barrier of humans for CWD has been studied using ultra-sensitive in vitro techniques. The first in vitro study suggested a substantial molecular barrier limiting susceptibility of humans to CWD [151]. Davenport et al. demonstrated positive seeding activity when human recombinant PrP was seeded with CWD, but not when using BSE, contradicting results observed in vivo [152]. Successful conversion of human PrP using different CWD seeds in PMCA has been reported by Barria et al. Their studies suggest that CWD from MM132 elk and CWD from reindeer have the highest potential to convert human PrP, followed by white-tailed deer prions and, finally, mule deer CWD isolates, which require an intermediate step of in vitro conditioning to deer substrate [153–155]. This positive conversion was achieved, however, in PMCA reactions using a large CWD prions-to- human substrate ratio [154].

## Conclusions

The prevalence and geographic spread of CWD continues to rise, expanding the likelihood of transmission to other species. Particularly of concern in North America is the risk to caribou, an endangered species. Although canids appear to have resistance to infection by CWD prions, other carnivores, i.e., the big felids, are predicted to be susceptible to infection. The zoonotic potential is still unclear but the increased prevalence of CWD in cervids will result in greater likelihood of human exposure.

## Abbreviations

BSE: bovine spongiform encephalopathy
CJD: Creutzfeldt-Jakob disease
CNS: central nervous system
CWD: chronic wasting disease
DMNV: dorsal motor nucleus of the vagus nerve
dpe: days post-exposure
ENS: enteric nervous system
GALT: gut-associated lymphoid tissue
PMCA: protein misfolding cyclic amplification
PrP^C^: cellular prion protein
PrP^CWD^: cervid PrP^Sc^
PrP^Sc^: pathological prion protein
RT-QuIC: real-time quaking-induced conversion
TME: transmissible mink encephalopathy
